# Factors Affecting Mistreatment of the Elderly in Long-Term Care Facilities

**DOI:** 10.3390/healthcare8030224

**Published:** 2020-07-23

**Authors:** Jeongmi Lim

**Affiliations:** Korea Institute for Health and Social Affairs, Sejong Special Self-Governing City 30147, Korea; jekljm@kihasa.re.kr; Tel.: +82-44-287-8255

**Keywords:** mistreatment, care worker, long-term care facilities, affecting factors

## Abstract

In long-term care facilities, elderly mistreatment occurs routinely and frequently. However, few studies have empirically explored the multifaceted risk factor of mistreatment. The purpose of this paper was to explore the factors affecting elderly mistreatment by care workers in Japanese long-term care facilities and to examine the relationship between these factors and mistreatment. This analysis was based on a sample of 1473 care workers from long-term care facilities and used multiple regression analyses. The results revealed that the nursing care level, work period, resilience, and attitude towards mistreatment among residents and staff were factors significantly associated with the degree of mistreatment. Facility size, an institutional environment that does not limit the behavior of residents, and family and community support for the elderly were among the institutional environment factors that had significant relationships with mistreatment. Staff gender, care-related qualifications, and workload were not associated with mistreatment. These findings suggest that strengthening the staff’s attitude and coping skills to prevent mistreatment, as well as interventions for changes in the institutional environment, are needed to prevent and reduce the prevalence of mistreatment in Japan. In addition, raising staff resilience to stress situations and building a resident-centered facility care environment is an important measure to reduce mistreatment.

## 1. Introduction

Elderly abuse and mistreatment are a commonly recognized human rights violation and a serious social problem in Japan. The mistreatment of elderly people can cause negative health problems such as disability, suicide attempts, and even death [1,2,3]. A record-high 621 cases of abuse in long-term care facilities for the elderly were reported in 2018 according to Japan’s Ministry of Health, Labour and Welfare [4]. Physical abuse was highest at 57.5%, followed by psychological abuse at 27.1%, neglect at 19.2%, financial exploitation at 5.8%, and sexual abuse at 5.4% [4]. The number of cases of abuse in facilities is based on reported and received cases, and it is likely that the actual number is much higher [5]. Moreover, as only 28.4% of the reported and received cases were judged to be abuse [4], approximately 70% of cases involving inappropriate and abusive behaviors were omitted from the abuse prevention and intervention policy. This phenomenon is similar in Korea [6]. Mistreatment and improper care is a serious issue, both in Japan and abroad.

The Elder Abuse Prevention and Caregiver Support Law of Japan defines elderly abuse as an abusive act perpetrated by a caregiver or another person to an elderly person aged 65 and over, and consists of physical abuse, psychological abuse, neglect, sexual abuse, and economic abuse. Although mistreatment is not included in the Japanese elder abuse prevention law, it can be regarded as abuse [7,8]. Mistreatment occurs routinely and frequently in facilities, and it also includes human rights abuse in gray areas where it is not clear whether they are abusive or not [7,8,9]. Mistreatment as abuse-related human rights violations includes inadequate facility environment, insufficient care, and behavior limitations of the elderly, and is reported to have negative effects on the physical and mental health of the elderly [7,8,9,10]. In addition, it is reported that abuse progresses gradually rather than suddenly [9]. It starts with abusive behavior that ignores the feelings and desires of the elderly and then escalates into serious abuse in the form of isolation and deprivation [9,11]. Mistreatment is a broader concept than elderly abuse, and can occur prior to, or at the same time as, elderly abuse [7,9]. In other words, although not legally included as abuse, mistreatment is equivalent to abuse; it can negatively affect the body and mind of the elderly and can also lead to serious abuse if it becomes routine or chronic [7,9,12]. Therefore, intervention that leads to the prevention and awareness of risks for mistreatment is a priority for the prevention of abuse. 

The current study is based on Schiamberg’s ecological model [13], which is a representative theory used to explain elderly abuse and illustrate the risk factors for elder abuse in long-term care facilities. According to this model, risk factors for mistreatment in nursing homes include patient traits, staff traits, institutional environments, and societal factors. In this study, Schiamberg’s model is employed as a theoretical framework. 

### 1.1. Resident Traits

Many studies indicate that cognitive and physical impairments in older adults and the elderly with dementia correlate with the highest risk of abuse [4,14]. Eighty percent of battered elderly individuals in Japanese nursing homes required a high nursing care level [4], and dementia was positively related to aggressive care worker behavior [4,14]. Mistreatment of the elderly in long-term care facilities increases with resident age [4]. 

### 1.2. Staff Traits

The majority of perpetrators were male workers [4,15], and mistreatment was related to their level of education and nursing care skills [16,17]. There are no consistent findings between period of work and mistreatment. For example, some studies reported that care staff (e.g., care worker, social worker) who had been working at the facility for a longer period were less likely to behave violently towards the elderly [16]. Other studies reported that a shorter work period increases abusive behavior [18]. Abusive behaviors were closely associated with a high workload and levels of work stress [15,19]. Previous studies conducted a survey of care workers working in a nursing home or group home to determine the relationship between the work environment factors and care workers’ awareness of mistreatment and abusive behavior [17,20]. These surveys found that worker awareness of mistreatment was associated with abusive behavior and mistreatment [17,20]. For example, some of the perpetrators in care settings did not know that their behaviors, such as embarrassing and restricting the behavior of the elderly, and restraining the elderly, were wrong, and thus they did not consider changing their behavior [18,21]. Resilience relates to a variety of characteristics, including self-efficacy, coping skills, and emotional control [22,23]. Resilient long-term care nurses were more likely to report a higher quality of care [24]. Abusive behaviors were related to poor care staff resilience and the inability to control their emotions [25]. Care staff (e.g., nurse, care worker) with high resilience are also less likely to behave violently towards the elderly because resilience buffers the symptoms of “burn out” [25,26]. 

### 1.3. Features of Institutional Environments

Mistreatment was associated with a large number of patients and facility size [4,14,18]. This is because the larger the number of residents, the more difficult it is to provide meticulous and private care. Work overload is a risk factor of burnout and mistreatment in long-term care facilities [19]. Previous studies have shown that there is a higher risk of mistreatment on nights with fewer staff and less supervision [7]. The lack of care staff increases the workload, which in turn increases inadequate care against the elderly [7,15]. In this regard, an increase in the number of night shifts is a risk factor for the occurrence of abuse. The number of staff working in the Japanese long-term care setting has been in insufficient supply every year since 2013, and the shortage is expected to be approximately 60,000 care staffs per year [27]. Previous studies reported that the lack of training was related to mistreatment by care workers, and care staff who lacked education showed higher levels of abusive behavior when providing care [19,20]. Thus, it is important that the institutional environment can provide the training and education necessary for sustained skill development. The lack of autonomy among care staff has been related to institutional rules and facility directors’ attitudes [28,29]. Findings show that facilities with low levels of work autonomy have a low quality of service [29,30]. Family and community support of older residents has been reported to reduce the isolation of older adults and to increase facility openness and mitigate the incidence of abuse [7,13]. Limiting the behavior and decision making of the elderly in geriatric care are human rights violations similar to abusive behavior [7,8,10]. An institutional care environment and organizational culture that does not restrict the behavior of the elderly can prevent mistreatment from occurring [7,9,10]. It is of note, however, that the majority of abuse and mistreatment studies have been conducted in the community and not in institutional settings [19,20]. This trend is similar in Japan. Little research has examined the abuse and mistreatment occurring in Japanese long-term care facilities. Mistreatment studies in institutional settings are difficult to conduct because of institutional refusal and resistance to surveys [5]. Although there have been studies presenting the incidence of institutional abuse and mistreatment [14], they did not empirically analyze the relationships among risk factors. Several studies have analyzed the relationship between abuse and resident characteristics [31], and the relationship between mistreatment and the staff or institutional environment [20], but only at the level of analyzing the relationships between each factor separately. In addition, previous studies do not adequately consider factors such as care worker resilience and institutional environmental characteristics (e.g., work autonomy, morale recognition, openness of the facility), which are considered important for improving the quality of care and preventing mistreatment. Mistreatment is influenced by both the internal characteristics of residents and care workers as well as factors such as the environmental characteristics of the facility, which should be considered influencing variables. The purpose of this study was to explore the factors affecting the mistreatment of the resident by the care workers and to examine the relationship between the risk factors and mistreatment. 

## 2. Materials and Methods 

### 2.1. Study Design and Participants

For this study, a questionnaire survey was conducted among 6000 care workers working in long-term care facilities registered in the Japanese Long-Term Care Insurance Business Database (WAMNET). The survey was conducted from 1 to 19 October 2015. A stratified two-stage random sampling was employed as the survey method. Each of the 1000 facilities were selected at equal intervals by region from 7667 facilities with 10 or more people and 12,589 facilities with 9 or fewer people nationwide. The questionnaire package (e.g., the study’s description form, participant’s inclusion criteria, consent form, questionnaire) and return envelopes were sent to the director of the selected facilities. We asked them to randomly select three care workers for each facility based on the inclusion criteria of participant. Participants eligible for the survey were limited to care workers who have been directly caring for residents for more than 3 months, because we thought they needed at least 3 months of caring experience to understand all the aspects of institutional settings and mistreatment. In addition, if care was not provided directly or no items were answered in the questionnaire, they were excluded from the analysis. The purpose of the study was explained to the questionnaire participants through the enclosed study description form and questionnaire package. The staff who agreed to participate in the survey were asked to return the questionnaire directly and anonymously. Finally, 1473 surveys were analyzed.

### 2.2. Ethical Approval and Procedures

For this study, the consent of the ethics committee to which the researcher belongs was obtained (IRB No. 15036). The study was explained to the research participants and consent forms were obtained. We did not send any reminders to the participants to return the questionnaire because the survey was conducted anonymously. Each of the subjects was assured anonymity and that the research findings would only be used for research purposes.

### 2.3. Measurement

As mentioned above, the risk factors in this study were categorized into three types: resident, staff, and institutional environment traits as Table 1.

#### 2.3.1. Resident Traits

The nursing care level is a comprehensive indicator to assess the care-recipients’ functional impairments and cognitive impairments. It is reported that the higher the nursing care levels of the residents, the greater the stress and exhaustion of the staff [14,31]. Nursing care levels range from grades 1 to 5, with higher grades indicating higher care requirements. In this study, the respondents were asked to indicate the average nursing care levels of the residents living in the facility to which they belong.

#### 2.3.2. Staff Traits

Staff traits included gender, the total work period as a care staff, and whether they had any care-related qualifications. Care worker resilience was measured using the resilience scale [32]. The scale includes 21 items that assess optimism (e.g., I feel like everything will work out in the end in some way), control (e.g., I try to understand the thoughts and pain of the resident), sociability (e.g., I have always been very good at getting to know people), behavioral skills (e.g., I think I am a tenacious person, I am able to finish what I decide to do), problem-solving ability (e.g., when an unpleasant event/conflict happens in the workplace, I gather information to solve the problem; when a resident displays aggressive behavior, I seek help from a care worker who has already experienced this problem), understanding of the psychology of others (e.g., I am good at noticing the feelings and subtle changes in the facial expressions of residents), and self-understanding of stress (e.g., I have a good understanding of how my thoughts and feelings change when hard things happen). Each item was rated with a four-point Likert-type answer format ranging from 1 = disagree to 4 = agree. Scores are summed, with higher scores indicating a higher tendency for resilience. In this study, Cronbach’s α = 0.898. The mistreatment scale (Cronbach’α = 0.966) [8] was revised and used based on the advice of researchers studying elderly abuse and field experts. Seven experts reviewed this and made minor adjustments. To test the face validity, we conducted a pilot study of 20 nursing staff (e.g., care worker, nurse) in long-term care facilities. 

The attitudes towards the mistreatment questionnaire consisted of 21 items related to four domains: lack of dignity, lack of exchange, role restriction, and lack of autonomy. The attitudes toward the mistreatment questionnaire included such items as “Resident can’t live the way they want and their wishes cannot be fulfilled” and “Force residents to participate in facility events or activities.” Each item was rated on a 4-point scale from 1 = not abuse at all to 4 = clear abuse. Scores are summed, with a higher score indicating high levels of awareness of the problem of mistreatment (Cronbach’s α = 0.929). In order to unify the respondents’ awareness, the questionnaire stated that abuse as defined in this survey referred to an act that infringes on the human rights of the elderly and causes serious stress or injury to the mind and body, regardless of the definition in the Elder Abuse Prevention Law. 

#### 2.3.3. Features of Institutional Environments

Large facilities with a large number of patients are associated with a higher incidence of mistreatment. The respondents chose one response, “yes” or “no”, regarding the facility size (1 = “facilities with 9 or fewer residents,” 0 = “facilities with 10 or more residents”). In order to measure the workload, the number of care worker night shifts per month was assessed. Five items developed by a previous study [33] were used to measure the level of job autonomy. The job autonomy questionnaire includes such items as “The workplace does not strongly demand that you follow a method of care that you think to be inappropriate” and “In the workplace, my opinions about resident care are reflected in work.” Each item was rated with a 4-point Likert-type answer format ranging from 0 = not implemented at all, to 4 = sufficiently implemented. The scores were summed, with a higher score indicating a higher autonomy at work (Cronbach’s α = 0.738). The institutional care environment questionnaire was selected by referring to the studies [8,34] to assess the level of the facility’s care performance that does not limit the behavior of residents. It consists of three items that examine the institutional care environment (e.g., “If the resident’s behavior is unavoidably restricted in the facility, it is strongly required to comply with the rules of the facility.”). Each item was rated on a 4-point scale from 1 = completely disagree, to 4 = completely agree. The scores were summed, with a high score indicating the high levels of an institutional care environment that does not limit the behavior of residents (Cronbach’s α = 0.717). The family and community support questionnaire was composed to assess the residents’ support from the family and community [34]. The family and community support questionnaire includes such items as “The workplace provides the care environment for family members to participate in the lives of residents and support for outings” and “The workplace provides the care environment that allows for daily connection between residents and community members.” Six items were rated on a 4-point scale, from 1 = not applicable to 4 = applicable. The scores were summed; the higher the score, the greater the support for the resident from the family and community (Cronbach’s α = 0.659).

#### 2.3.4. Mistreatment Behavior

Mistreatment behavior was measured using the Caregiver Mistreatment Scale [8]. Twenty-one items (e.g., “Resident can’t live the way they want and their wishes cannot be fulfilled” and “Force residents to participate in facility events or activities”) were rated with a four-point Likert-type answer format ranging from 1 = never behave this way, to 4 = often behave this way. This consists of the same twenty-one items as the scale of attitudes toward mistreatment. Scores were summed, with higher scores indicating a higher tendency toward abusive behavior (Cronbach’s α = 0.926).

### 2.4. Data Analysis

Data were analyzed using SPSS for Windows 21. (SPSS Inc., Chicago, IL, USA). Descriptive statistics were used to examine the characteristics of the sample. Prior to conducting the multiple regression analysis, the risk factors were tested to see if the variables were linearly independent of each other. In this sample, the values of the VIF (variance inflation factor) were less than two, which indicated that there was no risk of multicollinearity. A hierarchical multiple regression analysis was conducted to understand the effects of risk factors on mistreatment in nursing homes.

## 3. Results

### 3.1. Sample Characteristic

Table 2 presents the characteristics of the study variables. First, the nursing care levels of the residents averaged 3.5. Most of the respondents were female (69.9%), Second, the average work period in long-term care was 9.2 years (standard deviation (SD) = 5.62), and 73% of care staff had care-related qualifications. The average number of night shifts per month was 4.6 (SD = 2.0). The mean score for resilience was 60.47 (SD = 8.94, range = 24–84), which indicates a slightly high tendency toward care worker resilience, and the attitude score toward mistreatment was 64.95 (SD = 10.22, range = 21–84). Third, the size of the facilities surveyed was 53.7% with fewer than 10 people and 46.4% with 10 or more people. The mean score of job autonomy of 12.66 (SD = 2.01, range = 4–16) indicates a slightly high tendency toward the level of autonomy at work. The mean score of the facility’s care performance level that does not limit the behavior of residents of 9.97 (SD = 1.63, range = 3–12) indicates a relatively high tendency toward a facility care performance degree that does not limit the behavior of residents. The mean score of resident support from the family and the community was 12.22 (SD = 2.59, range = 4–16). Finally, the mean score of mistreatment of 39.43 (SD = 9.34, range = 21–84) indicates a relatively low tendency toward mistreatment behaviors.

### 3.2. Factors Affecting Mistreatment

A hierarchical regression analysis was conducted to analyze the factors affecting mistreatment in nursing homes, and the results are shown in Table 3. Model 1 examined the relationship between resident factors (residents’ nursing care level) and the degree of mistreatment (F = 38.0, *p* < 0.001). This model explained 2.5% of the variance of mistreatment (*p* < 0.001). The residents’ nursing care level (β = 0.159, *p* < 0.001) was statistically associated with mistreatment. The severity of mistreatment was more likely to increase as the nursing care level increased. In model 2, staff factors were added to model 1. This model examined the care workers’ gender, work period, qualifications related to care, resilience, and attitudes toward mistreatment behavior (F = 18.6, *p* < 0.001). Model 2 explained 33.3% of the variance of mistreatment (*p* < 0.001), and the R square (0.333) significantly increased (*p* < 0.001). Resilience (β = −0.425, *p* < 0.01) and attitudes to mistreatment (β = −0.278, *p* < 0.001) were significantly related to the level of mistreatment. It was found that the lower the resilience of care workers (β = −0.269), the more mistreatment occurred. Less mistreatment was reported by care workers with a high problem attitude toward mistreatment.

In model 3, institutional environment factors were added to model 2 (F = 227.7, *p* < 0.001). The R square (0.466) significantly increased (*p* < 0.001). The results showed that the nursing care level (β = 0.060, *p* < 0.05), work period in long-term care (β = −0.052, *p* < 0.05), resilience (β = −0.277, *p* < 0.001), attitudes towards mistreatment (β = −0.218, *p* < 0.001), size of facility (β = −0.056, *p* < 0.05), autonomy at work (β = −0.120, *p* < 0.001), institutional care environment that does not limit the behavior of residents (β = −0.182, *p* < 0.001), and the family and community support for residents (β = −0.179, *p* < 0.001) were significantly associated with mistreatment. The number of night shifts per month was not a significant predictor in this model, however. Specifically, the larger the facility, the more mistreatment occurred. Autonomy at work, an institutional care environment that does not limit the behavior of residents, and family and community support for residents statistically lessened the severity of mistreatment among older adults. 

## 4. Discussion

The purpose of this study was to identify the factors affecting the mistreatment of the resident by the care workers in Japanese long-term care facilities. The findings showed that the nursing care level, staff work period, resilience, attitude towards mistreatment, facility size, autonomy at work, institutional care environment that does not limit the behavior of residents, and family and community support for residents were statistically associated with mistreatment. In addition, these variables accounted for 46.6% of mistreatment behavior. 

Our results showed that care workers who have been working at a facility for a shorter period are more likely to behave violently towards residents. These results are consistent with those of a previous study [16], reporting that limited employee work experience is related to mistreatment. This means that the shorter the work experience, the less understanding of inappropriate or abusive behavior, which can increase the likelihood of mistreatment. This also implicates that earlier intervention (e.g., before inappropriate care becomes routine) might efficiently prevent care workers from abusing residents. Strategies are needed to increase the level of understanding of inappropriate behavior in new employees with a short history of working in facilities, and to increase human rights sensitivity at the same time. 

Gender was not associated with mistreatment. This may be because the majority (approximately 70%) of respondents were female, so the association with mistreatment could not be accurately verified. There was no significant relationship between the presence and absence of a qualification related to care, workload (e.g., number of night shifts per month), and mistreatment. This means that mistreatment occurs regardless of qualifications and workload. This is in contrast to previous studies that reported that staff without professional training and education [17,18] and high workloads [20] are more likely to behave violently toward the elderly. These results have several implications. As many of the participants in this study were qualified, it is possible that a significant relationship between qualifications and mistreatment was not demonstrated. Although obtaining a care-related qualification does not prevent mistreatment, the effects of education can emerge when education and training are provided in a timely and appropriate manner to reduce mistreatment. Therefore, practice-oriented education and skill training for mistreatment prevention and mitigation needs to be provided in the curriculum for care-related certifications. The number of night shifts was not associated with mistreatment. This may be because the majority (approximately 88%) of respondents worked less than six night shifts per month, so the association with mistreatment could not be accurately verified. Future research is needed to verify the relationship between these variables. On one hand, facility size was related to mistreatment. Facility size is related to the number of residents living in the facility, and the more people living there, the more often the mistreatment can occur. This is because more residents means more workload and more stressful situations for care workers. However, as we have reviewed in the relationship between the number of night shifts and abusive behavior, an increase in workload does not immediately imply an increase in abusive behavior. To develop a specific mistreatment prevention strategy, it would need further investigation regarding the relationship between workload (e.g., facility size, the number of shifts, staff to resident ratio) and mistreatment.

Resilience was the most influential variable for mistreatment. The results are similar to those of a previous study which demonstrated that resilience alleviates job stress and improves service quality [24,26]. Therefore, in order to prevent the incidents of mistreatment, above all, strategies to increase the resilience of employees are required. In particular, resilience as defined in this study is composed of the ability to understand and cope with stressful situations such as relationship conflicts in the workplace or the physical and linguistic violence of residents. Therefore, the development of educational programs that enhance the ability to cope with these stressful situations and the strengthening of training will be effective in preventing mistreatment. In addition, in order to improve the understanding of other peoples’ psychology, which is another component of resilience, training on the health status of the elderly and cultivating specific care practice skills to respond to the mood and minor facial expression changes of the elderly is needed. The recognition of mistreatment severity reduced the occurrence of mistreatment and had the greatest influence after resilience. This means that the lower the problem perception of mistreatment, the more likely it is that mistreatment will occur, and that it is very useful to strengthen the problem perception of improper care to prevent mistreatment. This is consistent with the findings that mistreatment behavior is only reduced when there is an understanding of mistreatment [9,20]. Therefore, in order to prevent mistreatment, it is first necessary to clarify the concept of mistreatment, which although not included in Japanese elder abuse prevention law, negatively affects the physical and psychological health of the elderly. Furthermore, education to achieve increased awareness of mistreatment is needed. There is a possibility that mistreatment occurs because employees are not sure how mistreatment affects the elderly. To prevent this, it is necessary to train employees about the negative effects of inappropriate care. Specifically, it is necessary to develop practical skills and strategies to share improper care-related cases and to address it through a case review meeting.

When job autonomy increases, mistreatment decreases, which is consistent with the finding that ensuring autonomy for care leads to improved service quality [29,30]. An institutional environment that not only recognizes the autonomy and professionalism of employees in elderly care, but also does not compel them to follow inappropriate instructions, is needed. An institutional care environment that does not limit the behavior of residents had a positive effect on mistreatment reduction. This means that how a facility’s care environment and philosophy surrounding elderly care is constructed can increase or prevent mistreatment. In order to prevent mistreatment, establishing a facility ideology and rules related to resident behavioral limitations, and sharing them with the entire staff, is needed. The greater the family and community support for the elderly, the less mistreatment occurs. This is an empirical verification of the claims that opening facilities to third parties can prevent inappropriate care and abuse [34]. In fact, several studies recommend third-party evaluation or the introduction of the ombudsman system as an abuse-prevention strategy [4,14]. This is aimed at reducing abuse cover-up mechanisms by increasing the openness of facilities. Family and community support and supervision systems should be established to realize high-quality care. The establishment of a system that provides opportunities for the elderly to not be isolated and to provide an opportunity for employees to have their care behavior checked by a third party is needed to prevent abuse.

Finally, this study is expected to provide basic data to establish strategies for prevention and intervention in abuse by exploring the factors affecting mistreatment that may escalate into serious abuse. In addition, we believe that our result provides understanding about the possible influencing factors for resident mistreatment by care workers in Japanese long-term care facilities. However, this study has several limitations. Since this study used variables excluding missing values for analysis, careful consideration of the results’ interpretation is needed, and it is limited in the analysis of causality in that it is a cross-sectional study. Further research should consider a longitudinal design that examines how risk factors affect mistreatment and abuse over time. The data of the current study were also collected by self-reported response from the respondents. Thus, data concerning mistreatment behaviors may have been reduced and under-reported. The facility director selected the survey respondents directly and distributed the questionnaire. Thus, the sample obtained may not be representative of the care worker in Japan. Because of the limited research, some important risk factors were missing from the model. There is a need to examine additional risk factors influencing mistreatment behavior. Future research should also examine whether the stress and burnout of care staff have any influence on mistreatment.

## 5. Conclusions

To prevent mistreatment and abuse, the measures to reduce the risk factors of mistreatment and to increase buffering factors are needed. First, it would be useful to take measures to increase the resilience of staff and to strengthen the awareness of problems related to mistreatment. Second, changes to facility environments (e.g., institutional care environment that does not limit the behavior of residents, strengthening family and community interaction) that enable better coping skills were more important in preventing mistreatment than simply reducing workloads and increasing education. Third, effective preventive measures should be simultaneously prepared in terms of the personnel level of care workers and the organizational level of the facility. For example, to prevent mistreatment, it may be more effective to strengthen the various management abilities of facilities as well as efforts to improve the coping ability of employees with stress situations (e.g., resident’s aggressive behavior, unpleasant event/conflict), since abuse and mistreatment is the structural result of a combination of diverse factors.

## Figures and Tables

**Table 1:** Detailed information about the variables and measurements.

Variables	Range of Answer Options
Resident traits	
Nursing care level	(1) 1 level–(5) 5 level
Care worker traits	
Gender	(0) female–(1) male
Work period in long-term care	year
Qualifications related to care	(0) no–(1) yes
Resilience	(1) disagree–(4) agree
Attitudes toward mistreatment	(1) not abuse at all–(4) clear abuse
Features of institutional environments	
Number of night shifts per month	month
Size of facilities	(0) facilities with 10 or more residents–(1) facilities with 9 or fewer residents
Autonomy at work	(0) not implemented at all–(4) sufficiently implemented
Institutional care environment	(1) completely disagree–(4) completely agree
Family and community support	(1) not applicable–(4) applicable.
Mistreatment behavior	(1) never behave this way –(4) often behave this way

**Table 2:** Characteristics of the variables.

Variables	Categories	*n*	%	M	SD
Resident traits					
Nursing care level				3.5	0.8
Care worker traits					
Gender	male	443	30.1		
	female	1030	69.9		
Work period in long-term care	<3 years	173	11.7		
	3–4 years	200	13.6		
	5–9 years	481	32.7		
	≤10 years	619	42		
Qualifications related to care	have	1075	73		
	none	398	27		
Resilience				60.47	8.94
Attitudes toward mistreatment				64.95	10.22
Features of institutional environments					
Number of night shifts per month				4.6	2.0
Size of facilities	≤9 or fewer	791	53.7		
	≥10 or more	682	46.4		
Autonomy at work				12.66	2.01
Institutional care environment				9.97	1.63
Family and community support				12.22	2.59
Mistreatment behavior				39.43	9.34

**Table 3:** Hierarchical regression analysis of factors affecting mistreatment (*n* = 1473).

Variables	Model 1 95% CI		Model 2 95% CI		Model 3 95% CI	
	β	t (*p*)	β	t (*p*)	β	t (*p*)
Nursing care level	0.159	6.163 (<0.001)	0.106	4.895 (<0.001)	0.060	2.453 (0.014)
Gender			0.006	0.288 (0.773)	−0.010	−0.495 (0.620)
Work period in long-term care			−0.021	−0.878 (0.380)	−0.052	−2.462 (0.014)
Qualification related to care			−0.004	−0.187 (0.852)	−0.009	−0.413 (0.680)
Resilience			−0.425	−19.200 (<0.001)	−0.277	−12.859 (<0.001)
Attitudes towards mistreatment			−0.278	−12.634 (<0.001)	−0.218	−10.945 (<0.001)
Size of facility					−0.056	−2.202 (0.028)
Number of night shifts					0.034	1.747 (0.081)
Autonomy at work					−0.120	−4.849 (<0.001)
Institutional care environment					−0.182	−7.740 (<0.001)
Family and community support					−0.179	−7.165 (<0.001)
Adj. R2	0.025		0.333		0.466	
F (*p*)	37.983 (<0.001)		18.564 (<0.001)		227.734 (<0.001)	

## References

[1] Dong X., Simon M.A. (2013). Association between reported elder abuse and rates of admission to skilled nursing facilities: Findings from a longitudinal population-based cohort study. Gerontology.

[2] Ogata M., Ago K., Ago M., Nakashima H., Ikematu K., Kondo T., Kita T., Tanaka N. (2007). Elder abuse: An autopsy case of suicide attributable to physical and financial abuse. Acta Criminol. Med. Leg. Jpn..

[3] Yunus R., Hairi N.N., Choo W.Y. (2019). Consequences of elder abuse and neglect: A systematic review of observational studies. Trauma Violence Abus..

[4] Ministry of Health, Labour and Welfare. https://www.mhlw.go.jp/content/12304250/000491671.pdf.

[5] Yamada H. (2008). What can be read from a survey of elderly abuse?. Jpn. J. Geriatr. Psychiatry.

[6] Ministry of Health and Welfare, Korea Elder Protection Agency (2019). Elder Abuse Status.

[7] Sibao K. (2008). Status and response of elderly abuse in the facility. Jpn. J. Geriatr. Psychiatry.

[8] Lim J. (2014). Trial to “Redefinition of elderly abuse” based on the recognition of elderly abuse of the nursing care staff: Focusing on the structure and features of "quasi-abuse". Jpn. J. Soc. Welf..

[9] Lim J. (2017). A newly proposed concept framework for elderly abuse based on the international classification of functioning, disability and health (ICF). Jpn. J. Soc. Welf..

[10] Malmedal W., Ingebrigtsen O., Saveman B.I. (2009). Inadequate care in Norwegian nursing homes: As reported by nursing staff. Scand. J. Caring Sci..

[11] Conrad K.J., Iris M., Ridings J.W., Rosen A., Fairman K.P., Anetzberger G.J. (2011). Conceptual model and map of psychological abuse of older adults. J. Elder Abus. Negl..

[12] Takasaki K., Sibao K., Hirata A. (2012). Improper care leads to abuse. Hureai Care.

[13] Schiamberg L.B., Barboza G.G., Oehmke J., Zhang Z., Griffore R.J., Weatherill R.P., Levente von H., Post L.A. (2011). Elder abuse in nursing homes: An ecological perspective. J. Elder Abus. Negl..

[14] Sendai, Tokyo and Ohu Centers for Dementia Care Research and Practices (2008). Report on Support for Abuse Prevention of Elderly People at Facilities.

[15] Arens O.B., Fierz K., Zúñiga F. (2017). Elder abuse in nursing homes: Do special care units make a difference? A secondary data analysis of the Swiss Nursing Homes Human Resources Project. Gerontology.

[16] Nakashima T., Kurata Y., Takigyti M., Okabe Y., Cho C. (2010). Study of prevention of elderly abuse from the perspective of improvement within care services: Analysis of the survey targeted at care worker. Res. J. Care Welf..

[17] Lim J. (2018). The effects of job environment on elderly maltreatment of care worker in long-term care insurance of Japan. J. Welf. Aged Inst..

[18] Natan M.B., Lowenstein A., Eisikovits Z. (2010). Psycho-social factors affecting elders’ maltreatment in long-term care facilities. Int. Nurs. Rev..

[19] Shinan-Altman S., Cohen M. (2009). Nursing aides’ attitudes to elder abuse in nursing homes: The effect of work stressors and burnout. Gerontologist.

[20] Matumoto N. (2015). Verification of the protective factors of mistreatment in group-living for dementia: A questionnaire survey of care staffs. J. Jpn. Soc. Dement. Care.

[21] Gibbs L.M., Mosqueda L. (2004). Confronting elder mistreatment in long-term care. Ann. Long Term Care.

[22] McCann C.M., Beddoe E., McCormick K., Huggard P., Kedge S., Adamson C., Huggard J. (2013). Resilience in the health professions: A review of recent literature. Int. J. Wellbeing.

[23] Hart P.L., Brannan J.D., De Chesnay M. (2014). Resilience in nurses: An integrative review. J. Nurs. Manag..

[24] Williams J., Hadjistavropoulos T., Ghandehari O.O., Malloy D.C., Hunter P.V., Martin R.R. (2016). Resilience and organisational empowerment among long-term care nurses: Effects on patient care and absenteeism. J. Nurs. Manag..

[25] Conlie Shaw M.M. (1999). Nursing home resident abuse by staff: Exploring the dynamics. J. Elder Abus. Negl..

[26] Song J., Huh S. (2018). Moderating effects of self-efficacy and social support on the relationship between resilience and burnout: Focusing on nurses’ experiences. Health Soc. Welf. Rev..

[27] Ministry of Health, Labour and Welfare. https://www.mhlw.go.jp/content/12201000/000363270.pdf#search=%27%E4%BB%8B%E8%AD%B7%E4%BA%BA%E6%9D%90%E4%B8%8D%E8%B6%B3+%E5%8E%9A%E7%94%9F%E5%8A%B4%E5%83%8D%E7%9C%81%27.

[28] Mrayyan M.T. (2004). Nurses’ autonomy: Influence of nurse managers’ actions. J. Adv. Nurs..

[29] Ichigawa W. (2000). Institutional Abuse: Why do Nurse Aids Behave to Abuse?.

[30] Currie V., Harvey G., West E., McKenna H., Keeney S. (2005). Relationship between quality of care, staffing levels, skill mix and nurse autonomy: Literature review. J. Adv. Nurs..

[31] Matumoto N. (2020). A study on the effect of residents’ factors which increased the risk, and the countermeasures, of institutional abuse. J. Jpn. Soc.Dement. Care.

[32] Hirano M. (2010). A study of the classification of resilience factors: Development of the bidimensional resilience scale (BRS). Jpn. J. Personal..

[33] Kuroda K., Okamoto H. (2004). A Study on the Development of Service Quality Evaluation Criteria and Evaluation Methods in Elderly Care Facilities.

[34] Nakasima K., Nakamura K. (2005). Practice Method for “Life Support” to Bring up Care Worker.

